# Exosomes Derived from M2 Macrophages Exert a Therapeutic Effect via Inhibition of the PI3K/AKT/mTOR Pathway in Rats with Knee Osteoarthritic

**DOI:** 10.1155/2021/7218067

**Published:** 2021-12-10

**Authors:** Zha Xi Da-wa, Ma Jun, Liu Chao-Zheng, Yang Sen-Lin, Lu Chuan, Li De-chun, Dong Zu-Nan, Zhao Hong-tao, Wei Shu-qing, Pei Xian-wei, Liu Wenbo, Li Ke-wen

**Affiliations:** ^1^Qinghai University Affiliated Hospital, Department of Joint Surgery, China; ^2^Qinghai University Affiliated Hospital, Department of Spine Surgery, China; ^3^Hebei PetroChina Central Hospital, China; ^4^Suzhou Municipal Hospital of Anhui Province, Department of Orthopedics, China; ^5^Translational Research Institute of Intensive Care Medicine, School of Anesthesiology, Weifang Medical University, China

## Abstract

Macrophages are commonly classified as M1 macrophages or M2 macrophages. M2 macrophages are obtained by stimulation of IL-4 with anti-inflammatory and tissue repair effects. Exosomes are 30–150 nm lipid bilayer membrane vesicles derived from most living cells and have a variety of biological functions. Previous studies have shown that macrophage exosomes can influence the course of some autoimmune diseases, but their effect on knee osteoarthritis (KOA) has not been reported. Here, we analyze the roles of exosomes derived from M2 macrophage phenotypes in KOA rats. Exosomes were isolated from the supernatant of M2 macrophages and identified via transmission electron microscopy (TEM), Western blotting, and DLS. The results showed that M2 macrophage exosomes significantly attenuated the inflammatory response and pathological damage of articular cartilage in KOA rats. In addition, a key protein associated with KOA including Aggrecan, Col-10, SOX6, and Runx2 was significantly increased, while MMP-13 was significantly suppressed following treatment with M2 macrophage exosomes. The present study indicated that M2 macrophage exosomes exerted protective effects on KOA rats mainly mediated by the PI3K/AKT/mTOR signal pathway. These findings provide a novel approach for the treatment of KOA.

## 1. Introduction

Knee osteoarthritis (KOA) is a degenerative disease with the main symptoms of knee joint pain and swelling, severely affected and accompanied by joint deformities [1]. Due to changes in people's dietary structure and increasing social aging, the incidence of osteoarthritis is increasing year by year [2]. Epidemiological investigations show that the incidence of osteoarthritis is about 40.2% [3]. At present, the most common treatment methods for osteoarthritis include physical therapy, drug therapy, surgical treatment, and traditional Chinese medicine treatment [4]. But there are side effects of medications prescribed in medicine and injury of surgical treatment. Therefore, there is an urgent need for new strategies to suppress the inflammatory effect and alleviate KOA.

Macrophages are one of the most common defensive immune cells in the body and play a vital role in the occurrence and development of inflammation-related diseases [5]. Macrophages involved in the synovial membrane coordinate inflammation and proliferation by secreting inflammatory factors and matrix metalloproteinases (MMPS) and play a key role in regulating the pathogenesis of osteoarthritic [6, 7]. Under in vitro culture conditions, M1 macrophages are induced by IFN-*γ*, and LPS and M2 macrophages are induced by Th2 type cytokines (IL-4, IL-13) [8]. M1 plays a proinflammatory effect, and M2 plays an anti-inflammatory effect [9]. M2 can also act on surrounding cells through paracrine function, improve the biological activity of vascular endothelial cells and fibroblasts, and participate in the process of tissue repair, scar formation, and injury healing. The biological functions of M2 macrophages may be related to their secreted exosomes [10, 11]. Since the discovery of exosomes in 1979, it was considered as “junk” metabolized by cells during development. With the continuous advancement of biotechnology, researchers have discovered that is a carrier for cells to exert their biological activities [12]. The specific nutritional factors released by exosomes have the effects of anti-inflammatory, immune regulation, restoring the homeostasis of the exosome, and promoting cartilage regeneration [13]. Moreover, the exosomes can repair articular cartilage, improve KOA symptoms, and delay disease progression [14]. However, the research of M2-exo in the treatment of inflammation-mediated diseases has not been reported.

The PI3K/AKT pathway is a signal pathway closely related to knee osteoarthritis [15]. PI3K/AKT is a classic anti-apoptotic pathway and plays a vital role in rapid signal transduction from the membrane to the nucleus. It can regulate the downstream pathways Bad, Caspase, and NF-*κ*B against osteoarthritis cartilage apoptosis [16]. The PI3K/AKT signaling pathway can regulate a variety of cell proliferation, apoptosis, and differentiation. This pathway is the most important to regulate chondrocyte proliferation, apoptosis, and matrix remodeling [17]. In the process of chondrocyte apoptosis, it plays a prominent role, and the PI3K/AKT signaling pathway is activated in various ways in the early stage of osteoarthritis [18]. Therefore, the present study was conducted to observe the protective effects of M2 macrophage exosomes on the proinflammatory factors and synovial tissues in the KOA rat model by treating KOA with M2 macrophage-derived exosomes, exploring the intervention effect of exosomes on PI3K/AKT/mTOR signaling pathway, and aiming to provide a basis for clinical treatment of KOA.

## 2. Experimental Part

### 2.1. Animals and Grouping

Eight weeks (male, 200-220 g) SD rats and knee osteoarthritis (KOA) rats used in this study were all purchased from the Animal Center of Qinghai University. All rats are kept in separate cages without pathogens, and they can eat and move freely. All animal experiments were carried out under the relevant regulations for the management and protection of experimental animals from Qinghai University. The animal experimental program has been approved and filed by the Experimental Animal Welfare and Ethics Committee of Qinghai University. 32 experimental animals were divided into a blank group (SD rat, Blank), KOA rat model group (Model), M2 macrophage treatment group (Model+M2-cell), M2 macrophage exosome treatment group (Model+M2-exo), and treatment group (*n* = 8).

### 2.2. Cell Isolation, Identification, Culture, and Treatment

Take the rat's femur and tibia, cut both ends, open the bone cavity, inject sterile PBS with a 10 mL syringe to flush out the bone marrow, add red blood cell lysate to resuspend, and lyse on ice for 10 minutes. After resuspending in complete medium, M-CSF (10 ng/mL, Peprotech) was used to induce monocytes to differentiate into macrophages, cultured for 7 days, replaced with a complete medium containing 10 ng/mL IL-4 (Peprotech, USA), and induced differentiation for 48 h; then, M2 macrophages was obtained.

### 2.3. Flow Cytometry

Phenotypic changes in macrophages were verified by flow cytometry using the BD LSRFortessa flow cytometer. After blocking FcRs (14-0161-82, eBioscience), cells were washed and resuspended in PBS supplemented with 1% heat-inactivated FBS and 0.01% NaN3. For CD206 staining, cells were fixed and permeabilized with a BD Cytofix/Cytoperm Fixation/Permeabilization Solution Kit (BD Biosciences, Franklin Lakes, NJ). Cells were then incubated with the FITC-CD206 antibody (GTX43682, genetex) for 30 min at 4°C in the dark. Flow cytometry analyses of cocultures were accomplished by nonenzymatic dissociation of cells from the culture plates. The macrophage cell suspension was gated to identify M2 macrophages cells using RFP fluorescence versus side scatter.

### 2.4. Exosome Isolation and Identification

M2 macrophages were cultured for 48 h under serum-free conditions, and the cell culture supernatant was collected. Dead cells were removed by high-speed centrifugation. The cells were then centrifuged at 10,000g for 30 min to remove cell debris, and the supernatant was collected at 150,000g for 90 min to collect M2-exo. The pellets were washed with PBS to remove contaminating proteins and centrifuged again at 150,000g for 90 min. Morphology of exosomes was examined by a transmission electron microscope (TEM), and the particle size was analyzed by Nanosight (DLS, Zetasizer Nano, Malvern Instruments). The TEM assay was performed as follows: exosomes were fixed overnight in 2% paraformaldehyde solution, centrifuged at 150,000g for 30 min and then suspended in anhydrous ethanol. l.2 *μ*L was added drop-wise onto the copper grid, dried, and assayed by TEM. Exosomes were resuspended with PBS (10 *μ*L PBS/1 mL culture media). After being resuspended, the exosomes stored exosomes at −80°C for later use.

### 2.5. Animal Experiment

Experimental mice were treated after acclimatization feeding. In the M2 macrophage exosome treatment group, mice received 50 *μ*L exosome suspension via the tail vein twice a week for 10 weeks. In the M2 macrophage treatment group, we prepare serum-free M2 macrophages resuspended in PBS, and mice received 50 *μ*L M2 macrophage suspension via the tail vein twice a week for 10 weeks. The blank group and model group received saline treatment for the same duration and frequency.

### 2.6. Elisa Assay

Elisa kits were used to detect proinflammatory-related factors in a joint tissue such as IL-1*β*, IL-6, and TNF-*α* (Shanghai Enzyme-linked Biotechnology Co., Ltd., China). The tissue supernatant was diluted to 100 *μ*L (1 : 20), following the manufacturer's instructions, incubated under the specific capture antibody and detection antibody, and finally read the absorbance value at 450 nm.

### 2.7. H&E Assays

The articular cartilage tissue was fixed with 4% neutral formaldehyde solution, dehydrated with concentration gradient alcohol, transparent with xylene, embedded in paraffin, sliced, and stained with H&E (Yeasen, China). Observe the histopathology under a microscope, take pictures, and save.

### 2.8. Van Gieson Assays

Paraffin sections were routinely dewaxed to water, stained with hematoxylin for 5-10 minutes and washed with water for 1 minute. Mix acid fuchsin and picric acid in a ratio of 1 : 9 to prepare a VG dyeing solution (Solarbio Life Science, China), and dye for 30-60 seconds. Direct differentiation with differentiation solution for 10 seconds (dehydrated, transparent, and sealed with neutral gum).

### 2.9. TUNEL Alexa Fluor 488 Staining

Tissue sections were dewaxed in xylene for 5-10 minutes. Switch to fresh xylene, and dewax for another 5-10 min. Treat with anhydrous ethanol for 5 min, 90% ethanol for 2 min, 70% ethanol for 2 min, and distilled water for 2 min. Add 20 *μ*g/mL DNase-free Proteinase K dropwise, and act at 20-37°C for 15-30 min. Then, wash 2 times with PBS. Add 50 *μ*L TUNEL (AAT Bioquest, China) detection solution to the sample, and incubate for 60 min at 37°C protected from light. The excitation wavelength range used was 450-500 nm, and the emission wavelength range was 515-565 nm (green fluorescence).

### 2.10. Immunohistochemical Assay

The articular cartilage tissue was fixed with 4% neutral formaldehyde solution, dehydrated with a concentration gradient of alcohol, transparent with xylene, and paraffin-embedded. The paraffin sections were routinely dewaxed to water, cooled at room temperature after antigen repair, sealed with 10% goat serum, dripped with diluted first antibody (Biolegend, Germany), overnight at 4°C, and incubated with the biotinylated second antibody at room temperature for 30 min, then DAB (3,3′-diaminobenzidine) color. Hematoxylin was added to stain again, then neutral tree gum was used to seal, and the images were observed and collected by a BA200 Digital Tertiary Camera Microscope.

### 2.11. Reverse Transcriptase-Polymerase Chain Reaction (qRT-PCR)

Total RNA was extracted from the cell and tissue samples using the Total RNA isolation Kit (Takara, RR9109) according to the manufacturer's instructions. cDNA synthesis was performed using a PrimeScript RT reagent Kit (Takara, RR047A) following the manufacturer's instruction. qRT-PCR was performed using the A PIKORed 96 (ThermoFisher, USA) with primers (All are listed in follows) using the SYBR Green™ Premix Ex Taq™ II (Takara, RR820A) following the manufacturer's instruction. The expression of each gene was first normalized to that of *β*-actin and was presented as a fold change by calculating the average expression level of each of the three samples divided by that of the controls at the same time point. The primer sequences are as follows: CD206-F: 5′-CTCTAAGCGCCATCTCCGTT-3′, CD206-R: 5′-ATGATCTGCGACTCCGACAC-3′; Arginase-F: 5′-CCAGTATTCACCCCGGCTAC3′, Arginase-R: 5′-ACAAGACAAGG TCAACGCCA-3′; PI3K-F: 5′-GCGTCAAGATCAGCTTATCCTT-3′, PI3K-R: 5′-TTTGTACTCGTGGCTAACA CC-3′; AKT-F: 5′-ATCATGAGC GATGTTACCA-3′, AKT-R 5′-TGAGGTTTCTCTTTATAGC CTA-3′; mTOR-F: 5′-AGAGGACCAGCAGCACAAGCAGGAG-3′, mTOR-R: 5′-GCAGTGGT GGTGGCATTGGTGATGTT-3′; and *β*-actin-F: 5′-CCGTAAAGACCTCTATGCCAACA-3′, *β*-actin-R: 5′-GCTAGGAGCCAGGGCAGTAATC-3′.

### 2.12. Western Blot

After treatment of M2-exo, the articular cartilage tissue from different groups was collected for Western blotting. Then, each sample was lysed with 60 *μ*L RIPA regent added with 1% thioglycol. Then, boil the sample in 100°C water for 5 minutes, and put in the refrigerator at -20°C. 5 *μ*L of each sample was separated by sodium dodecyl sulfate-polyacrylamide gel electrophoresis (SDS-PAGE) and transferred to polyvinylidene difluoride (PVDF) membranes. Subsequently, membranes were blocked for 1 h at room temperature and then blotted with the primary antibody at 4°C overnight, PI3K/p-PI3K (1 : 1000, Abcam), AKT/p-AKT (1 : 500, Absin), mTOR/p-mTOR (1 : 500, Shenggong BBILIFE), CD9 (1 : 500, Abcam), CD63 (1 : 500, Abcam), and CD81 (1 : 500, Abcam), followed by 1.5 h incubation with horse-radish peroxidase- (HRP-) conjugated anti-rabbit secondary antibody at room temperature. Finally, chemiluminescence was quantitated with a Versadoc Imaging System.

### 2.13. Statistical Analysis

Experiments were performed at least three times as indicated. Graphprism 6.0 software was used in this research. The differences between the two groups were evaluated by a *T*-test, and between groups (>2) were evaluated by one-way analysis of variance (ANOVA). p value < 0.05 was considered statistically significant.

## 3. Results

### 3.1. Polarization of BMMs into M2 Type and Extraction and Identification of Exosomes

**Figure 1:**
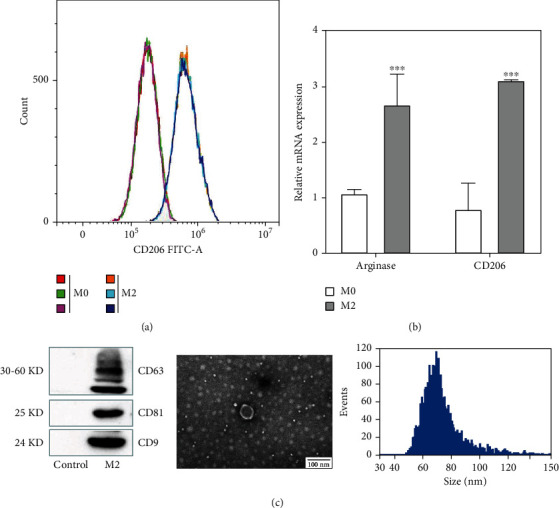
Identification of M2 type macrophages and exosomes. (a) Flow cytometric detection of CD206 expression in M0 and M2 type macrophages. (b) Relative expression of M2-type macrophage surface markers (Arginase and CD206). (c) Exosome signature protein identification, TEM detection of the morphological structure of M2-exo, and particle size distribution of M2-exo. *T*-test was used for statistical analysis; ^∗∗∗^*p* ≤ 0.0001.

Bone marrow-derived monocytes were isolated from mouse femurs and tibias and were polarized by M-CSF to M0-macrophages, followed by IL-4 to M2 macrophages. As shown in Figure 1(a), the fluorescence of CD206 protein in M0-type macrophages became significantly stronger after IL-4 stimulation, and about 80% of M0 was successfully polarized to M2-type. As shown in Figure 1(b), after IL-4 stimulation of M0 macrophages, the M2 macrophage markers Arginase and CD206 were significantly increased at the mRNA level (*p* ≤ 0.0001). The above experimental results showed that M2 macrophages were successfully polarized and could be used for exosome isolation. M2 macrophage-derived exosomes were observed to be spheroid morphology under transmission electron microscopy (Figure 1(c)). DLS showed the exosomes with a mean diameter of about 79.86 nm. The above experimental results indicate the successful isolation and extraction of M2 macrophage-derived exosomes, which can be used for subsequent ex vivo experiments.

### 3.2. Inhibition of Inflammatory Response in KOA Rats by M2-Type Macrophage Exosomes

**Figure 2:**
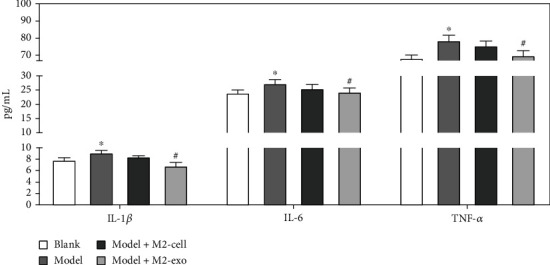
Effect of M2-type macrophage exosomes on proinflammatory factors in the joints of KOA rats. The experimental results are expressed as the mean ± standard deviation (SD); ^∗^*p* < 0.05, compared with the blank group; ^#^*p* < 0.05, compared with the model group.

To further investigate the local anti-inflammatory effect of M2-exo on joints, the proinflammatory cytokines in the joint tissues of each group of rats were examined by ELISA assay. As shown in Figure 2, compared with blank rats, the levels of IL-1*β*, IL-6, and TNF-*α* increased in the model group, and the mean concentrations of the three factors were 8.17 ± 0.12 pg/mL, 27.13 ± 0.32 pg/mL, and 81.32 ± 0.314 pg/mL, respectively. There was a slight decrease in the levels of the three proinflammatory factors in the M2-cell injection group. The concentrations of proinflammatory cytokines IL-1*β*, IL-6, and TNF-*α* in the joint tissues of rats in the M2-exo group were significantly reduced, and the mean concentrations of the three factors were 7.13 ± 0.21 pg/mL, 24.23 ± 0.22 pg/mL, and 76.12 ± 0.31 pg/mL, respectively (*p* < 0.05).

### 3.3. Effect of M2-Type Macrophage Exosomes on Articular Cartilage Repair in KOA Rats

**Figure 3:**
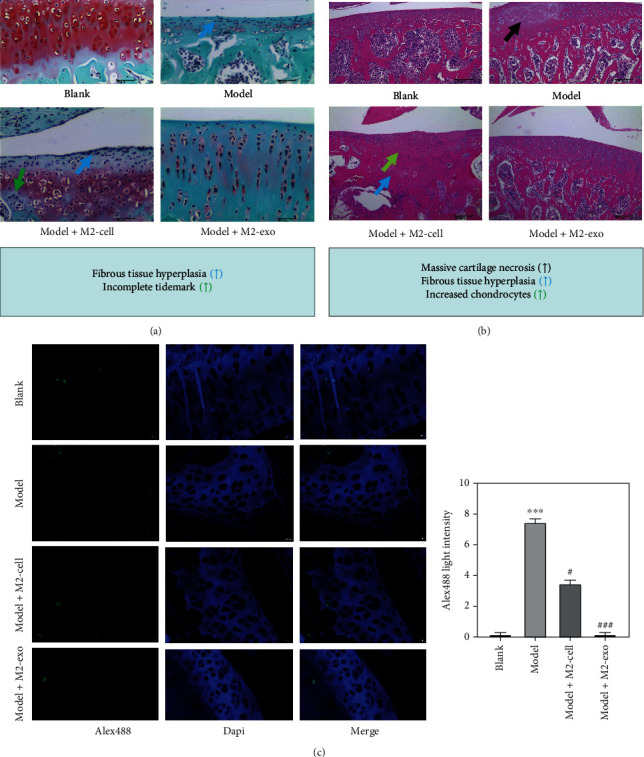
Histochemical detection of articular cartilage in KOA rats. (a) Van Gieson staining. (b) HE staining. (c) TUNEL staining. ^∗^*p* < 0.05 and ^∗∗∗^*p* < 0.0001, compared with the blank group. ^#^*p* < 0.05 and ^###^*p* < 0.0001, compared with the model group.

There is histochemical detection of M2 macrophage exosomes on the repair of articular cartilage in KOA rats. Van Gieson staining showed that the model group had a total defect of the cartilage layer with no tide lines and a large amount of fibrous tissue hyperplasia compared to the blank group. The Model+M2-cell group had a variable thickness of cartilage layers, superficial defects of articular cartilage, hyperplasia of fibrous tissue, and incomplete tide lines. However, under M2-exo treatment, all these responses disappeared, consistent with the blank group (Figure 3(a)). The results of HE staining showed that, compared with the blank group, the model group had erosion of articular cartilage, localized extensive necrosis, loss of chondrocytes, a large amount of fibrous-like material exuding from the necrotic area, significant increase in the number of chondrocytes, and clusters of chondrocytes with the irregular arrangement. See Model+M2-cell articular cartilage erosion, uneven cartilage surface, and some chondrocyte necrosis. However, under M2-exo treatment, these responses disappeared, consistent with the blank group (Figure 3(b)). TUNEL staining showed that compared with the blank group, the model group had the strongest green fluorescence, and the M2 macrophage injection group had reduced fluorescence, while the intensity of green fluorescence in the M2-exo treatment group was almost the same as that of the blank group, which indicated that M2-exo had a certain protective effect on joint apoptosis (Figure 3(c)).

### 3.4. Effects of M2-Type Macrophage Exosomes on the Expression of Related Proteins in the Joint Tissue of KOA Rats

**Figure 4:**
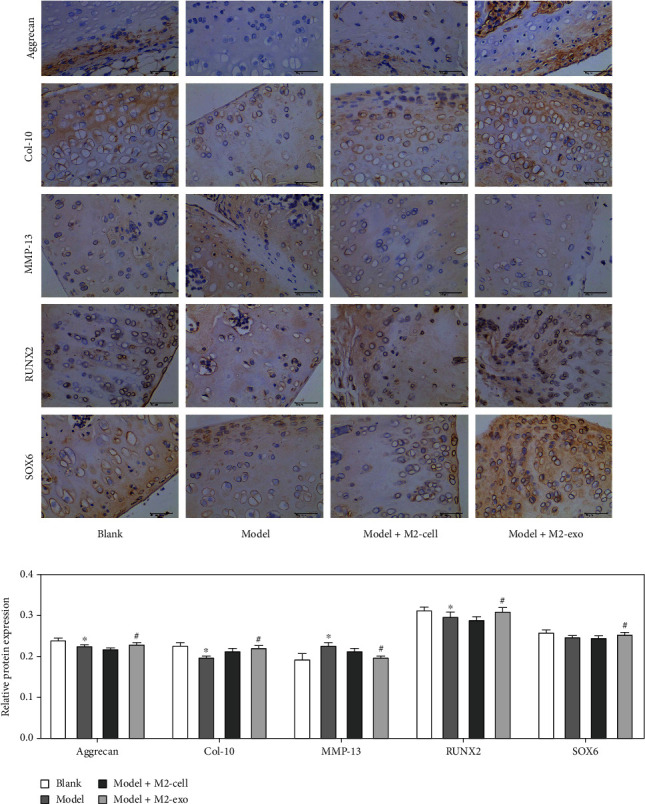
Expression of Aggrecan, Col-10, MMP-13, SOX6, and Runx2 proteins in joint tissues of KOA rats. ^∗^*p* < 0.05, compared with the blank group. ^#^*p* < 0.05, compared with the model group.

Immunohistochemistry was used to detect the changes of bone formation associated proteins. Compared with the blank group, the expression levels of Aggrecan, SOX6, and Runx2 decreased in the model group. Compared with the model group, Aggrecan, SOX6, and Runx2 increased in the M2-cell treatment group and M2-exo. However, compared with the blank group, the expression of MMP-13 was the highest in the model group and decreased in the other groups (Figure 4).

### 3.5. Effect of M2-Type Macrophage Exosomes on PI3K/AKT/mTOR Pathway in Joint Tissue of KOA Rats

**Figure 5:**
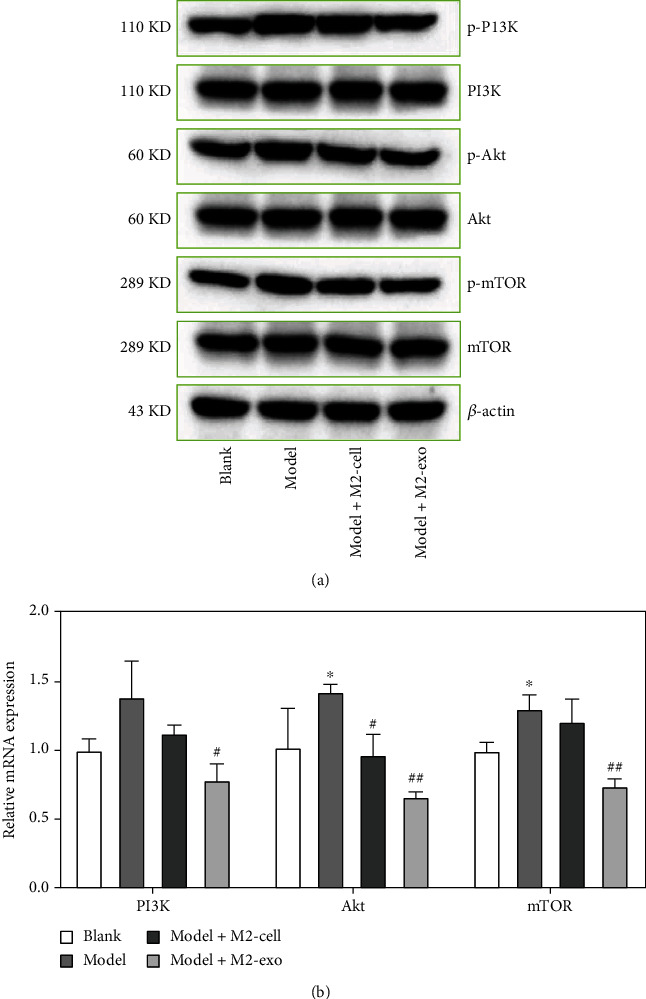
Expression changes of PI3K/AKT/mTOR pathway in rats with cartilage defects in plateau knee osteoarthritis. (a) PI3K/AKT/mTOR pathway Western blot assay results. (b) PI3K/AKT/mTOR pathway mRNA assay results. ^∗^*p* < 0.05, compared with the blank group. ^#^*p* < 0.05 and ^##^*p* < 0.001, compared with the model group.

To assess whether M2-exo regulated apoptosis and autophagy via PI3K/Akt/mTOR pathway, Western blot and qRT-PCR were used to verify this reaction. Western blot further explained the problem. Compared with that in the blank group, the expression level of PI3K/AKT/mTOR in the model group increased, but the M2-cell histone protein did not change significantly. These phosphorylated proteins were significantly reversed under M2-exo treatment (Figure 5(a)). As shown in Figure 5(b), compared with that in the blank group, the m RNA expression level of PI3K/AKT/mTOR in the model group increased, while the M2-cell treatment group had a certain downward trend. Compared with the model group, M2-exo significantly downregulated the expression of PI3K/AKT/mTOR, which was close to blank. The above experimental results indicate that PI3K/AKT/mTOR may be a potential pathway for M2-exo to treat cartilage damage.

## 4. Discussion

During the occurrence and development of KOA, the knee joint will cause damage to the synovium of the joints, increasing the permeability of the subsynovial capillary, so that a large amount of exudate will accumulate in the joint cavity, thereby causing joint swelling, pain, and trigger joint inflammation reactions [19]. Previous studies have suggested that excessive inflammation and cartilage degeneration are common events that occurred in osteoarthritis [14]. Persistent antigens will cause a continuous immune response in the body and are also the main factors involved in the pathogenesis [14, 20]. Therefore, reducing inflammation and protecting chondrocyte apoptosis is essential for the treatment of knee osteoarthritis patients.

Although the pathogenesis of KOA is not clear, the infiltration of inflammatory cells in the synovial membrane is considered the main cause of sustained occurrence [20]. Macrophages are important natural immune cells with the function of chemotaxis, phagocytosis, and secretion of various inflammatory mediators, which play an important role in immune defense [21]. It is well known that M2 macrophages can be activated by Th2 cytokine IL-4 [8]. In this study, 80% of bone marrow-derived monocytes were successfully activated by IL-4 to M2 macrophages. CD206 and Arginase were highly expressed in cells. Studies have shown that in many inflammatory tissues, M2 macrophages promote tissue repair by secreting cytokines. The biological function of M2 macrophages may be related to the secretion of exosomes. Studies proved that M2 macrophage-derived exosomes carry miR-1271-5p to regulate cardiac injury in acute myocardial infarction through downregulation SOX6 [22].

In recent years, many studies have reported that exosomes derived from different types of stem cells have the potential for regenerative medicine treatment. However, many studies simply add exosomes in damaged tissues and organs, and the detailed mechanism of tissue structure and function recovery is not very clear [23]. In this experiment, the exosomes of M2 macrophage cells were successfully isolated. The expression of the marker proteins CD9, CD81, and CD63 was identified, and the particle size was determined to be about 79.86 nm.

Studies have proven that exosomes derived from human mesenchymal stem cells can repair osteoarthritis cartilage degeneration [14]. At the same time, inflammatory factors are closely related to knee osteoarthritis. IL-6 and IL-1*β* are also the most important cytokines that destroy KOA articular cartilage and cartilage matrix degradation [24, 25]. The inflammation in the articular cavity caused by TNF-*α* leads to the regression of KOA joints [26]. After the intervention of M2-exo, the degree of local inflammation, namely, the three important proinflammatory cytokines TNF-*α*, IL-6, and IL-1*β*, showed that the inflammatory mediators were significantly reduced.

Recent studies have shown that cartilage destruction is the core pathological change of KOA, but synovial inflammation is also a vital manifestation in the process of KOA. In the KOA model, the synovial cell inflammatory response, with the cascade amplification effect, joint synovial hyperplasia, hyperemia, and joint effusion increase. However, with the progress of KOA, the synovial inflammation gradually stabilized, and cartilage destruction became the main pathological manifestation of KOA. In this study, we observed the expression and change of synovial inflammation in the process of KOA from the molecular biology level through animal experiments [27]. After the intervention of M2-exo, the articular cartilage structure was intact, the chondrocytes were evenly distributed, the tide line was intact, and there were no obvious cartilage defects or cartilage. Pathological changes such as cell necrosis and fibrosis did not show up.

Aggrecan, Col-10, MMP-13, SOX6, and Runx2 are closely related to osteoarthritis. Aggrecan is the main component of proteoglycan (extracellular matrix, ECM), which constitutes the unique tissue structure and mechanical properties of cartilage tissue [28]. Runx2 belongs to the family of transcription factors and can promote the maturation of osteoblasts [29]. MMP-13 is highly expressed in arthritis patients, which can effectively degrade various collagens, and has a certain damage effect on articular cartilage [30]. COL-10 is mostly distributed around cartilage cell clusters, which can lead to overmaturation of articular chondrocytes, osteogenesis in the cartilage, collagen degradation, and osteophyte formation [31]. SOX6 gene is involved in the coupling regulation of cartilage formation and osteogenesis [32]. Under M2-exo treatment, the expression level of Col-10, SOX6, and Runx2 increased, while the expression level of MMP-13 decreased. These osteogenesis-related proteins returned to levels close to those of the blank group, which indicated M2-exo has a therapeutic effect on knee osteoarthritis, but the mechanism is still unclear.

mTOR is a serine/threonine-protein kinase closely related to cell proliferation, cell growth, and cell differentiation [33]. It has been found that the upstream signaling pathways with mTOR as the junction mainly include the PI3K/Akt signaling pathway and AMPK/mTOR signaling pathway [34, 35]. Studies have confirmed that the PI3K/Akt/mTOR signaling pathway is mainly involved in the regulation of apoptosis and autophagy [36]. Current studies believe that the occurrence of KOA is the result of the activation of the PI3K/Akt/mTOR signaling pathway in the body [17]. In this study, the expression of phosphorylated protein and mRNA of PI3K, pAkt, and p-mTOR in the model group significantly increased, and M2-exo intervention can reduce the protein and mRNA expression of PI3K, Akt, and mTOR in KOA model rats. The result indicates that M2-exo may act as a negative regulator of mTOR, and the PI3K/Akt/mTOR signaling pathway may be a potential therapeutic pathway for KOA.

## 5. Conclusion

**Figure 6:**
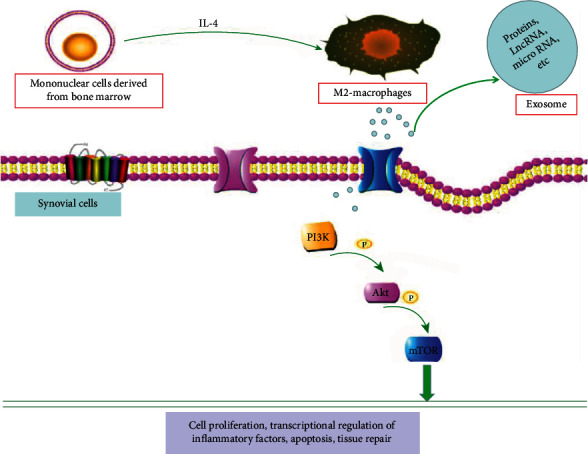
Schematic diagram of the protective mechanism of exosomes derived from M2 macrophage against KOA through the PI3K/AKT/mTOR signaling pathway. Exosomes derived from M2 macrophage can regulate the production of proinflammatory factors and the level of osteogenesis-related proteins.

This study preliminarily confirmed the protective effect of M2 macrophage-derived exosomes on KOA rats and further explained the potential signal pathways involved in bone protection (Figure 6). However, the current research is not in-depth enough for the specific mechanism of the exosomes. Exosomes are the main carriers of proteins, LncRNA, microRNA, etc. for regulating the biological behavior of cells. Further exploration of the substances that M2 macrophages-derived exosomes can regulate arthritis may provide new ideas for the evaluation and intervention of osteoarthritis.

## Data Availability

The datasets used or analyzed during the current study are available from the corresponding author on reasonable request.
